# Tyrosine Kinase Inhibitors as Antiangiogenic Drugs in Multiple Myeloma

**DOI:** 10.3390/ph3041125

**Published:** 2010-04-22

**Authors:** Domenico Ribatti

**Affiliations:** Department of Human Anatomy and Histology, University of Bari Medical School, Piazza G. Cesare, 11, Policlinico 70124, Bari, Italy; E-Mail: ribatti@anatomia.uniba.it; Tel.: +39-080-5478240; Fax: +39-080-5478310.

**Keywords:** angiogenesis, antiangiogenesis, multiple myeloma, tyrosine kinase inhibitors

## Abstract

Tyrosine kinase inhibitors are a new class of anticancer drugs, that are capable of directly interacting with the catalytic site of the target enzyme and thereby inhibiting catalysis. Therapeutically useful tyrosine kinase inhibitors are not specific for a single tyrosine kinase, but rather they are selective against a limited number of tyrosine kinases. The success of imatinib-mesylate (Gleevec^®^) for the treatment of patients with chronic myeloid leukemia has opened a intensive search for new small molecular compounds able to target other protein tyrosine kinases involved in the malignant transformation. This review article is focused on the use of tyrosine kinase inhibitors as antiangiogenic molecules in the treatment of multiple myeloma.

## 1. Introduction

Receptor tyrosine kinases (RTKs) are transmembrane proteins containing an extracellular lectin binding domain and an intracellular catalytic domain. Many of the processes involved in tumor growth, progression and metastasis are mediated by signaling molecules acting downstream from activated RTKs. 

Tyrosine kinase inhibitors are small molecules able to pass the plasma membrane [1]. The tyrosine kinase vascular endothelial growth factor receptors (VEGFRs) are crucial mediators in angiogenesis and stimulation of VEGFRs and other RTKs causes massive activation of signaling pathways in endothelial cells. Tyrosine kinase inhibitors inhibit not only VEGFRs but also other receptors in the superfamily of RTKs, including the platelet derived growth factor receptor (PDGFR). Inhibitors of VEGF signaling not only interfere with angiogenesis but also cause regression of some tumor vessels [2], causing changes in all components of the vessel wall of tumor, consisting in loss of endothelial cell fenestrations, regression of tumor vessels, and appearance of basement membrane ghosts [3].

In 2005, the Food and Drug Administration (FDA) granted regular marketing approval for sorafenib, a small and oral inhibitor for the treatment of patients with advanced renal cell carcinoma [4]. Sunitinib is a small oral multikinase inhibitor of VEGFR, PDGFR, *c-kit* and Flt-3 kinase activity [5]. Tyrosine kinase inhibitors can be taken orally, if necessary in a salt form of the inhibitor. For example, sunitinib is taken as sunitinib malate, while sorafenib as tosylate sorafenib.

Tyrosine kinase inhibitors can be subdivided in three categories. Type I kinase inhibitors recognize the active conformation of a kinase. An example is sunitinib, which demonstrates competitive inhibition to ATP agonist VEGFR-2 and PDGFR-β. Type II kinase inhibitors recognize the inactive conformation of a kinase. An example is sorafenib, which blocks the phosphorylation of VEGFR, PDGFR, *Raf* and *kit* by using a hydrophobic packet to indirectly compete with ATP. A third class of kinase inhibitors is known as “covalent” inhibitors and have been developed to covalently bind to cysteines at specific sites of the kinases. An example is vandetanib, which in addition to targeting VEGFR, inhibits epidermal growth factor receptor (EGFR) [6]. 

An advance in this field includes the development of soluble decoy receptor incorporating both VEGFR-1 and VEGFR-2 domains (VEGF-Trap), binding VEGF with higher affinity than previously reported VEGF antagonists [7]. The VEGF-Trap abolished mature, pre-existing vasculature in established xenografts resulting in almost completely avascular tumors subsequently followed by marked tumor regression and suppressed tumor growth [7].

## 2. Angiogenesis in Multiple Myeloma

In multiple myeloma bone marrow angiogenesis measured as microvascular density increases with progression from monoclonal gammopathy of undetermined significance (MGUS) to nonactive multiple myeloma and active multiple myeloma, and is related with the plasma cell labeling index [8]. Assuming that microvascular density depends on angiogenesis, these results are consistent with the notion that angiogenesis favors expansion of the multiple myeloma mass by promoting plasma cell proliferation [8].

Myeloma plasma cells induce angiogenesis directly via the secretion of angiogenic cytokines, such as VEGF and fibroblast growth factor-2 (FGF-2), and indirectly by induction of host inflammatory cell infiltration, and degrade the extracellular matrix with their matrix degrading enzymes, such as matrix metalloproteinase-2 and -9 (MMP-2 and MMP-9) and urokinase-type plasminogen activator [8]. 

Mosaic blood vessels consisting of endothelial cells, highly proliferative circulatory endothelial progenitors, haematopoietic stem cells, haematopoietic progenitor cells, macrophages, mast cells and tumor cells are recognizable [9,10,11]

More recently, we have carried out a comparative gene expression profiling of multiple myeloma endothelial cells and MGUS endothelial cells with Affymetrix U133A arrays [12]. Twenty-two genes were found differentially expressed (14 down-regulated and 8 up-regulated) at relatively high stringency in multiple myeloma endothelial cells compared with MGUS endothelial cells. Deregulated genes are mostly involved in extracellular matrix formation and bone remodeling, cell adhesion, chemotaxis, angiogenesis, resistance to apoptosis, and cell-cycle regulation. Validation was focused on *DIRAS3*, *SERPINF1*, *SRPX*, *BNIP3*, *IER3*, and *SEPW1* genes, which were not previously found to be functionally correlated to the overangiogenic phenotype of multiple myeloma endothelial cells. Small interfering RNA for three up-regulated genes (*BNIP3*, *IER3*, and *SEPW1*) affected critical multiple myeloma endothelial cell functions mediating the cell overangiogenic phenotype, that is proliferation, apoptosis, adhesion, and capillary tube formation.

Reciprocal positive and negative interactions between plasma cells and bone marrow stromal cells, namely hematopoietic stem cells, fibroblasts, osteoblasts/osteoclasts, chondroclasts, endothelial cells, endothelial cell progenitor cells, T cells, macrophages and mast cells, mediated by an array of cytokines, receptors and adhesion molecules, modulate the angiogenic response in multiple myeloma [13,14]. Since the cytokine network between plasma cells and bone marrow stromal cells in the bone marrow milieu promotes plasma cell growth, survival and migration, and plasma cells in the bone marrow are resistant to conventional agent treatment, targeting this network constitute a rationale to the treatment of multiple myeloma.

## 3. Tyrosine Kinase Inhibitors in the Treatment of Multiple Myeloma (Table 1)

Lin *et al.* [15] showed that vatalanib (PTK787/ZK222584), an orally administered broad-spectrum tyrosine kinase inhibitor of VEGFR-1, -2, -3, PDGFR-β, *c-kit*, inhibited proliferation and migration of multiple myeloma cells. Pandiella *et al.* [16] showed that imatinib mesylate (STI 571) blocked cell-cycle progression in multiple myeloma and potentiated the effects of conventional antimyeloma agents *in vitro*. However, in a phase II clinical trial in patients with refractory/relapsed myeloma, no response was obtained [17].

Zangari *et al.* [18] and Kovacs *et al.* [19] evalutated the activity of, respectively, SU5416 a small tyrosine kinase inhibitor of VEGFR-1, -2, -3 and of vandetanib (ZD6474) in patients with refractory multiple myeloma and observed a decrease in VEGF serum levels in patients with stable disease, but not objective response. Podar *et al.* [20,21] demonstrated that pazopanib (GW786034B) and GW654652, two broad-spectrum tyrosine kinase inhibitors of VEGFR-1, -2, -3, PDGFR, *c-kit*, inhibited *in vivo* multiple myeloma cell proliferation, migration and survival, VEGF-induced up-regulation of adhesion molecules on both endothelial and tumor cells, and exerted an antiangiogenic activity *in vivo*. However, a recent phase II clinical trial in 21 multiple myeloma patients treated with pazopanib, did not show any clinical response [22].

Ramakrishnan *et al.* [23] showed that sorafenib exerted a significant anti-myeloma activity and synergized with common anti-myeloma drugs. Coluccia *et al.* [24] has shown constitutive activation of PDGFR-β/*Src,* two dasatinib targets, in plasma cells and endothelial cells isolated from patients with multiple myeloma. Moreover, dasatinib significantly delayed multiple myeloma tumor growth and angiogenesis *in vivo*, showing a synergistic cytotoxicity with other anti-myeloma drugs, *i.e.* melphalan, prednisone, bortezomib, and thalidomide.

In about 10–20% of multiple myeloma patients, a translocation [t(4;14)] involving FGF receptor 3 (FGFR-3) is associated with poor prognosis [25,26,27]. Small molecules with selective tyrosine kinase inhibitory activity (SU5402, SU10991, PD173074, PKC412) have been validated in preclinical models of multiple myeloma [28,29,30]. 

**Table 1:** Kinase inhibitors in clinical trials for multiple myeloma.

Drug	Targets						Ref.
	VEGFR-1	VEGFR-2	VEGFR-3	PDGFR-β	*c-kit*	EGFR	
Dasatinib				×	×		[23]
GW654652	×	×	×	×	×		[20]
Imatinib mesylate				×	×		[15]
Pazopanib	×	×	×	×	×		[19]
Sorafenib	×	×	×	×			[22]
SU5416	×	×	×				[17]
Vandetanib	×	×	×			x	[18]
Vatalanib	×	×	×	×	×		[14]

## 4. Toxicities

As observed in patients with solid tumors, the most consistent side effect that has been observed with anti-VEGF agents is hypertension, which is usually manageable with medical therapy. Other serious but rare side effects include thromboembolic events, ischemic cerebrovascular accidents and congestive heart failure. Furthermore, bleeding complications and wound healing problems may be caused by a disturbance of the interaction of platelets with the vasculature.

## 5. Concluding Remarks

Starting from the first successful myeloma treatment in the late 1960s with a combination of melphalan and prednisone, treatment of multiple myeloma has changed substantially as a result of drug development.

The use of antiangiogenic agents is an exciting strategy for the treatment of multiple myeloma. However, several questions remain to be answered, including the most effective treatment strategies (e.g. combination with chemotherapy or other targeted agents, schedule and timing of administration) and the long-term consequences of prolonged antiangiogenic therapy on normal tissues.

The main problem in the development of antiangiogenic agents is that multiple angiogenic molecules may be produced by tumors, and tumors at different stages of development may depend on different angiogenic factors for their blood supply. Therefore, blocking a single angiogenic molecule was expected to have little or not impact on tumor growth. Multi-targeted kinase inhibitors, or a combination of inhibitors, may target additional angiogenic pathways, carry out a broader efficacy and avoid resistance. 
